# Comparison of Rapid and Automated Antigen Detection Tests for the Diagnosis of SARS-CoV-2 Infection

**DOI:** 10.3390/diagnostics12010104

**Published:** 2022-01-04

**Authors:** Dorian Petonnet, Stéphane Marot, Isabelle Leroy, Julien Cohier, Charline Ramahefasolo, Safietou Mansaly, Vincent Calvez, Anne-Geneviève Marcelin, Sonia Burrel

**Affiliations:** 1 AP-HP, Hôpital Pitié-Salpêtrière, Service de Virologie, 75013 Paris, France; petonnet.dorian@gmail.com (D.P.); stephanesylvain.marot@aphp.fr (S.M.); isabelle.leroy@aphp.fr (I.L.); julien.cohier@aphp.fr (J.C.); charline.ramahefasolo@aphp.fr (C.R.); safietou.mansaly@aphp.fr (S.M.); vincent.calvez@aphp.fr (V.C.); anne-genevieve.marcelin@aphp.fr (A.-G.M.); 2 Institut Pierre Louis d’Epidémiologie et de Santé Publique (IPLESP), Sorbonne Université, INSERM U1136, 75646 Paris, France

**Keywords:** COVID-19, SARS-CoV-2, antigen testing, manual assay, automated assay, nucleocapsid, diagnostic, nasopharyngeal swab

## Abstract

SARS-CoV-2 viral antigen detection may be an interesting alternative to RT-PCR for the diagnosis of SARS-CoV-2 infection as a less laborious or expensive method but requires validation. This study aimed to compare the performance of the DiaSorin™ LiaisonXL automated quantitative antigen test (QAT) and the AAZ™ rapid antigen test (RAT) to the DiaSorin™ MDX RT-PCR assay. A total of 242 nasopharyngeal samples were tested at La Pitié-Salpêtrière University Hospital (Paris, France). Performances for the detection of variants of SARS-CoV-2 were further investigated. RATs were visually read for qualitative results and band intensity was determined. Overall sensitivity was 63.2% for QAT and 58.6% for RAT. For RT-PCR Ct value 25, sensitivity was 89.8% for both tests. Both tests showed comparable sensitivity for detection of variants. There was a strong relationship between antigen concentration and band positivity. On the same set of samples these tests share similar performances.

## 1. Introduction

Coronavirus disease 2019 (COVID-19) has led to more than 5.3 million deaths worldwide since December 2019 (WHO Coronavirus COVID-19 Dashboard, update 20th December 2021) [1]. Early diagnosis of new cases is a key element in pandemic control strategies. Viral RNA detection in nasopharyngeal samples (NPS) through real-time RT-PCR is considered as the gold standard method for the diagnosis of SARS-CoV-2 infection, however it is not always applicable for mass detection. Viral nucleocapsid (N) protein is a highly conserved 46 kDa protein composed of 422 amino acids which participate in viral RNA packaging and forms the ribonucleoprotein core. It is an essential component of betacoronavirus and the most abundantly produced viral protein during SARS-CoV-2 infection [2,3]. Viral N protein detection may be an interesting alternative to RT-PCR as a more accessible, faster and affordable method but requires further validation. Indeed, a reliable automated antigen assay, which has good performances on respiratory samples, could be taken into consideration for mass testing. This study aimed to compare the detection performance of SARS-CoV-2 N protein in NPS samples using the automated quantitative antigen test (QAT) LIAISON^®^ SARS-CoV-2 Ag assay (DiaSorin, Saluggia, Italy) and the rapid antigen test (RAT) COVID-VIRO^®^ (AAZ, Boulogne-Billancourt, France) compare to the Simplexa™ COVID-19 Direct RT-PCR assay on the LIAISON^®^ MDX instrument (DiaSorin).

## 2. Methods

### 2.1. Study Design

A total of 87 positive and 155 negative left-over NPS were prospectively and consecutively selected among fresh routine NPS samples collected from healthcare workers (HCWs) tested at La Pitié-Salpêtrière University Hospital (Paris, France) between 1 December 2020 and 18 January 2021. HCWs were symptomatic or asymptomatic, but detailed clinical data were not available for most of the individuals. Then, no analysis was performed regarding clinical information focusing only on the analytical performances of the assays. NPS samples were collected in universal viral transport media (VTM) recommended by manufacturers. After collection, samples were sent to the virology department for RT-PCR analysis. Different techniques were randomly used: Cobas^®^ SARS-CoV-2 (Roche Diagnostics, Branchburg, NJ, USA), Simplexa™ COVID-19 Direct (DiaSorin, Saluggia, Italy), BioFire^®^ SARS-CoV-2 (BioMerieux, Salt Lake City, UT, USA), and NeumoDX^®^ SARS-CoV-2 (QIAgen, Hilden, Germany). After the first round of analysis, samples were stored for a maximum of 24 h between 2 and 8 °C before QAT and RAT processing according manufacturer’s instructions. Of note, RAT was tested upon all the positive samples but upon only 7 negative samples since specificity had already been assessed in previous studies.

### 2.2. RT-PCR

Samples were processed and analyzed according to manufacturer’s instructions. Prior to batch analysis, NPS specimens were inactivated at 56 °C for 30 min and stored at −80 °C for less than one week. RT-PCR was performed on 50 µL of preprocessed VTM using the Simplexa™ COVID-19 Direct RT-PCR assay on the LIAISON^®^ MDX instrument (DiaSorin). This RT-PCR assay targets both spike (S) and ORF1ab viral genes. The latter, ORF1ab gene target, was used as reference because of a better sensitivity. However, the crossing threshold (Ct) values were recorded for both targets (S and ORF1ab). Higher Ct values represent a low viral RNA load.

### 2.3. Quantitative Antigen Test (QAT)

NPS samples were inactivated by adding 500 µL of fresh VTM into a tube containing 500 µL of sample inactivation buffer (DiaSorin). To complete inactivation, tubes had to be kept at room temperature for 2 h before testing. Prior to batch analysis, inactivated samples were stored at −20 °C up to one week according to the manufacturer’s instructions.

N antigen quantification in inactivated samples was performed using the LIAISON^®^ SARS-CoV-2 Ag assay (chemiluminescent immunoassay (CLIA) technology) on the LIAISON^®^ XL instrument (DiaSorin). Results are expressed as 50% tissue culture infectious dose (TCID_50_) per mL. As recommended by the manufacturer, N antigen value ≥200.0 TCID_50_/mL is considered as a “positive” result, between 100.0 and 200.0 TCID_50_/mL as an “equivocal” result, and <100.0 TCID_50_/mL as a “negative” result. The limit of detection and upper limit of quantitation are 22.0 and 100,000.0 TCID50/mL, respectively. To analyze the results, QAT equivocal results were considered positive for performance comparison.

### 2.4. Rapid Antigen Test (RAT)

COVID-VIRO^®^ (AAZ), a membrane-based immunochromatography technology RAT, was performed according to the manufacturer’s instructions. The performances (sensitivity and specificity) of this RAT on VTM with adapted protocol were previously assessed in a French evaluation [4]. One hundred microliters of unprocessed VTM added to 100 µL of sample buffer supplied with the kit for optimal performance. One hundred microliters of the subsequent solution was dropped on RAT specific well. After 15 min of incubation time, results were read visually for qualitative interpretation. For quantitative results, band intensities were quantified using ImageJ^®^ tool (NIH). Background levels were determined and normalized to standardize measurements.

### 2.5. SARS-CoV-2 Variants Testing

The performances for the detection of SARS-CoV-2 historical B.1 lineage strain, SARS-CoV-2 variants of concern (VOCs) B.1.1.7 named “Alpha”, and SARS-CoV-2 VOC B.1.351 named “Beta” were further investigated from viral culture supernatants. Supernatants were successively diluted in a ratio of 1:10 and tested for both antigen detection and RT-PCR assays.

### 2.6. Statistical Analysis

Statistical analyses (Mann-Whitney or Fisher tests were used as appropriate) and figures were performed using Graphpad Prism v6. *p* value < 0.05 was considered as statistically significant. Correlation coefficient r were calculated with Spearman formula. Correlation coefficient r of >0.6 and >0.8 were interpreted as a “good” and as “strong” correlation, respectively.

The positive RT-PCR test results have been categorized and analyzed according to their quantitative ORF1ab RT-PCR Ct value: Ct ≤ 23, Ct ≤ 25, and Ct ≤ 33. Notably, Ct values ≤33 and ≤23 are the thresholds considered by the French Society of Microbiology as “compatible with significant viral excretion” and “compatible with high viral excretion”, respectively. Moreover, Ct value ≤ 25 was reported as a proposed threshold of transmissibility [4]. Then, QAT and RAT performances were determined across Ct threshold categories.

## 3. Results

**Figure 1:**
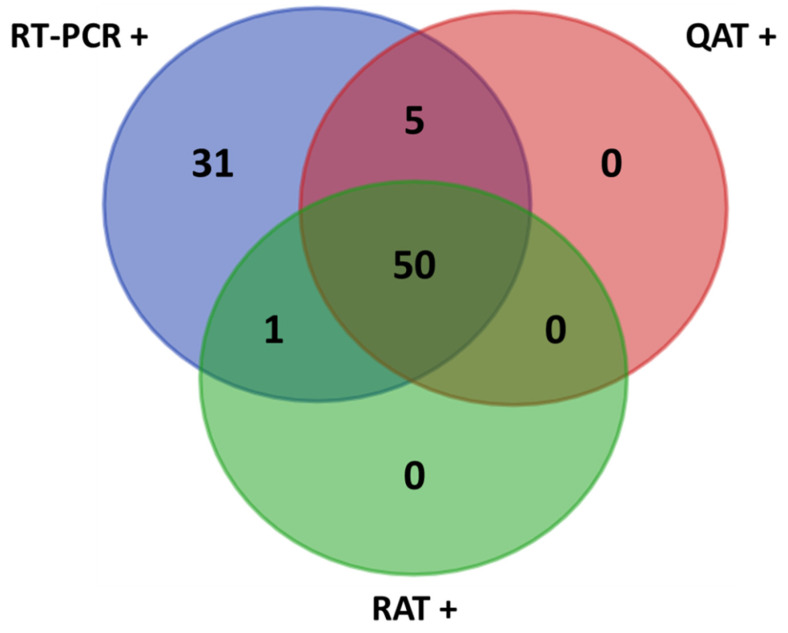
Venn diagram of the results. +: positive samples; QAT: quantitative antigen test; RAT: rapid antigen test; RT-PCR: reverse-transcription polymerase chain reaction.

**Figure 2:**
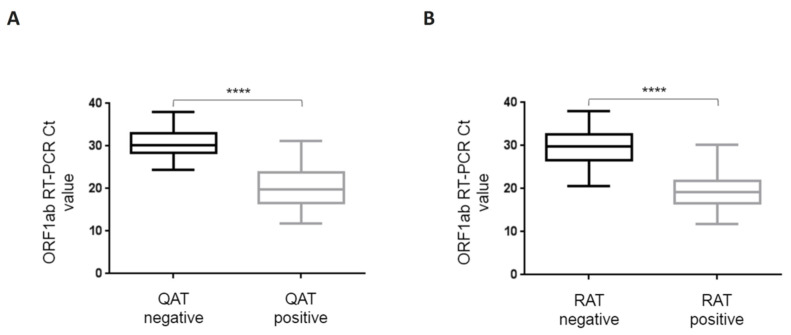
Box-and-whisker plots for ORF1ab RT-PCR Ct values depending on qualitative QAT (**A**) and RAT (**B**) results. ORF1ab RT-PCR Ct value is represented on the ordinate. QAT (**A**) and RAT (**B**) results are divided in two groups, positive (including equivocal) and negative. P value of the difference is represented on the graph as “****” for *p* < 0.0001. Boxes and whiskers show IQR and range of values (down to the smallest and up to the largest), respectively. Ct: crossing threshold; IQR: interquartile range; QAT: quantitative antigen test; RAT: rapid antigen test.

**Figure 3:**
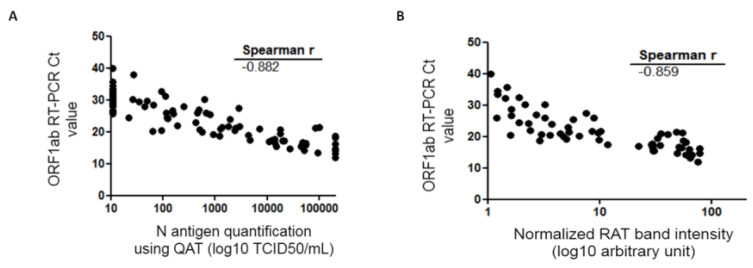
Spearman correlation of the QAT quantification (**A**) or RAT (**B**) and RT-PCR Ct value. QAT results are expressed as TCID_50_/mL (logarithmic scale) and represented on the abscissa (**A**). RAT quantitative results are expressed as band intensities (logarithmic scale) quantified using ImageJ^®^ tool after normalization and represented on the abscissa (**B**). ORF1ab RT-PCR Ct value is represented on the ordinate. Correlation coefficient r calculated with Spearman formula is shown on the graph. Ct: crossing threshold; N protein: viral nucleocapsid protein; QAT: quantitative antigen test; RAT: rapid antigen test; TCID_50_/mL 50% tissue culture infectious dose per milliliter.

**Figure 4:**
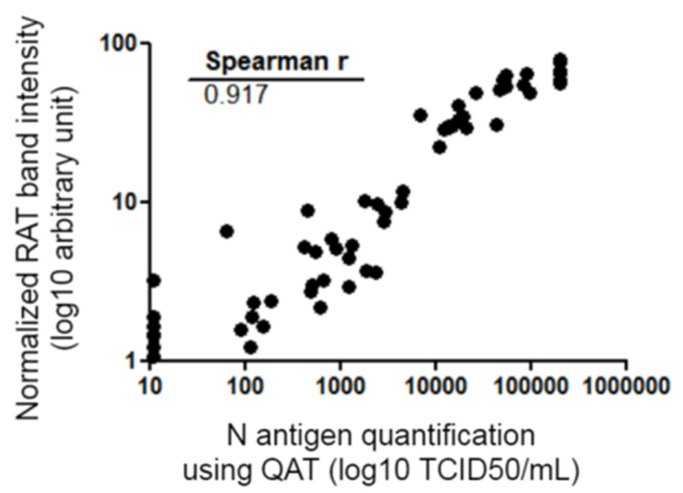
Spearman correlation of the quantitative data of RAT and QAT. QAT results are expressed as TCID_50_/mL (logarithmic scale) and represented on the abscissa. RAT quantitative results are expressed as band intensities (logarithmic scale) quantified using ImageJ^®^ tool after normalization and represented on the ordinate. Correlation coefficient r calculated with Spearman formula is shown on the graph. Ct: crossing threshold; N protein: viral nucleocapsid protein; QAT: quantitative antigen test; RAT: rapid antigen test; TCID_50_/mL 50% tissue culture infectious dose per milliliter.

The median age of the 242 participants was 57 years (interquartile range; IQR: 40–68), with a male/female ratio of 8/7. Overall, from the 87 positive NPS included in this study, the median Ct value of ORF1ab RT-PCR target was 20.8 (IQR: 16.6–26.8). S and ORF1ab RT-PCR Ct values showed a strong correlation (Spearman r = 0.983, (95% confidence interval, CI: 0.976–0.990)) (see Supplementary Figure S1). This study evaluated the performance of N antigen testing assays compared with the real time RT-PCR for SARS-CoV-2 detection: QAT vs. RT-PCR and RAT vs. RT-PCR. Moreover, this study allowed comparing QAT and RAT (QAT vs. RAT) to evaluate the potential added value of using an automated assay (QAT) over a manual assay (RAT) for antigen testing. The overall obtained results are presented below and in Table 1 and Table 2 and Figure 1, Figure 2, Figure 3 and Figure 4.

### 3.1. QAT vs. RT-PCR

Considering QAT, overall sensitivity compared to RT-PCR was 63.2% [95% CI: 53.1–73.4%] with a specificity of 100% [CI: 97.6–100%]. Indeed, 55 samples were considered “positive” with QAT, including seven with equivocal results. For ORF1ab RT-PCR Ct value ≤33, ≤25, and ≤23, sensitivity was 67.9% [CI: 57.7–78.1%], 89.8% [CI: 81.3–98.3%], and 91.1% [CI: 82.8–99.4%], respectively. No false positive results were found with QAT among the 155 RT-PCR negative samples (98.7% had a QAT result below the limit of detection, i.e., <22.0 TCID_50_/mL) (Table 1). For samples considered as “equivocal” with QAT, ORF 1ab RT-PCR median Ct value was 25 (IQR: 24.3–26.3). Among the 32 RT-PCR positive samples considered as “negative” with QAT, ORF1ab RT-PCR median Ct value was 30.1 (IQR: 28.5–32.8), while for the 55 RT-PCR positive considered as “positive” with QAT (including “equivocal”), ORF1ab RT-PCR median Ct value was 19.8 (IQR: 16.6–23.4). A Mann-Whitney test was used to compare median ORF1ab Ct values for RT-PCR positive samples whether they were “positive” or “negative” with QAT and found a statistically significant difference (*p* < 0.0001) (Figure 2A). There was a strong correlation between N antigen concentration (expressed as TCID_50_/mL) and OFR1ab RT-PCR Ct value (Spearman r = −0.883 [CI: −0.923; −0.823]) (Figure 3A).

### 3.2. RAT vs. RT-PCR

Considering RAT, overall sensitivity compared to RT-PCR was 58.6% [95% CI: 48.3–69.0%]. Indeed, 51 samples were considered “positive” with RAT. For ORF1ab RT-PCR Ct value ≤33, ≤25, and ≤23, sensitivity was 63.0% [CI: 52.4–73.5%], 89.8% [CI: 81.3–98.3%], and 93.3% [CI: 86.0–100%], respectively (Table 1). Among the 36 RT-PCR positive samples considered as “negative” with RAT, ORF1ab RT-PCR median Ct value was 29.7 (IQR: 26.7–32.5), while for the 51 RT-PCR positive considered as “positive” with RAT, ORF1ab RT-PCR median Ct value was 19.1 (IQR: 16.6–21.6). Mann-Whitney test was used to compare median ORF1ab Ct values for RT-PCR positive samples whether they were “positive” or “negative” with RAT and found a statistically significant difference (*p* < 0.0001) (Figure 2B). There was a strong correlation between band intensity and OFR1ab RT-PCR Ct value (Spearman r = −0.860 [CI: −0.907; −0.790]) (Figure 3B).

### 3.3. QAT vs. RAT

Antigen concentration measured using QAT and RAT band intensity were compared. There was a strong relationship between N antigen concentration (expressed as TCID_50_/mL) and band positivity (Spearman r = 0.917 [CI: 0.874–0.946]) (Figure 4). Performances of QAT and RAT using RT-PCR as the gold standard were compared. A Fisher test was used and no statistically significant difference was found [95% CI of difference: −0.099 to 0.191]; *p* = 0.64.

### 3.4. Detection of Variants Using QAT or RAT

Both tests showed comparable sensitivity for detection of SARS-CoV-2 Wuhan strain and Alpha and Beta SARS-CoV-2 VOCs (Table 2). For all viruses, negative detection of N antigen in cell culture supernatants is linked to the 1:1000 dilution: QAT signal were repeatedly <100.0 TCID_50_/mL and no band was visible with RAT. Moreover, ORF1ab RT-PCR Ct value of viral RNA detection on 1:1000 supernatants were 24.5 for Wuhan strain, 25.6 for Alpha VOC, and 23.6 for Beta VOC.

## 4. Discussion

Antigen assays are typically characterized by a lower sensitivity but a good specificity compared to RT-PCR for the diagnosis of SARS-CoV-2 infection. WHO recommended criteria of 80% sensitivity and 97% specificity for validating the suitability of an antigen assay compared to an approved nucleic acid amplification test (NAAT) (WHO SARS-CoV-2 antigen-detecting rapid diagnostic tests: An implementation guide) [5]. Multiple evaluations of the performance of different antigen tests are available in the scientific literature [6,7,8,9]. We aimed to prospectively evaluate N antigen detection in NPS using the quantitative high-throughput DiaSorin LIAISON^®^ SARS-CoV-2 Ag assay (designed as QAT) and the AAZ COVID-VIRO^®^ assay (designed as RAT) in comparison to viral RNA detection using gold-standard RT-PCR. In order to do this, fresh positive and negative NPS samples primarily screened by SARS-CoV-2 RT-PCR were randomly selected. As a whole, this study showed that the QAT and RAT used in this work share similar performance on the same set of NPS samples. As expected, these two antigenic tests showed keep-up performance over extended experiments on SARS-CoV-2 VOCs since N protein is highly conserved and that VOC mutations are mainly linked to the S protein (Table 2) with rare exceptions [10]. Next, overall sensitivity was 63.2% [CI: 53.1–73.4%] for QAT and 58.6% [CI: 48.3–69.0%] for RAT (Table 1). These results are comparable to previous reported data [4,8,11,12,13,14]. QAT and RAT in our study did not meet the WHO acceptance criterion for sensitivity. Nevertheless, antigen tests were found to show better results in NPS samples with lower RT-PCR Ct values (i.e., higher viral loads), as previously published. Indeed, both QAT and RAT sensitivity increased to >90% when ORF1ab RT-PCR Ct value ≤ 23 (Table 1). RAT and QAT negative results were mainly associated with ORF1ab RT-PCR Ct values > 25, which can be related to lower viral loads and potentially less infectiousness. Interestingly, our data showed that there is a strong correlation between antigen concentration determined by QAT, RAT band intensity, and RT-PCR Ct values (Figure 3 and Figure 4). Although correlation between antigen detection and infectivity is composite factors [15,16,17], our results could support the use of antigen assays as tools to rapidly identify individuals, whether they are asymptomatic or not, with significant viral excretion [9,18]. Despite limited sensitivity, the easy-to-perform antigen tests are of great interest where NAAT is unavailable, where prolonged turnaround time (TAT) precludes clinical utility or when it offers a cost-effective solution alternative to RT-PCR. Automated antigen assays have another advantage of high throughput screening possibility, however they had relatively long TAT compared to RAT [3,9,13,19]. Thereby, further strategies should therefore be evaluated to appropriately position these tools in the diagnostic context.

## Figures and Tables

**Table 1:** Comparison of QAT and RAT with RT-PCR.

	QAT vs. RT-PCR	RAT vs. RT-PCR
Overall Sensitivity	63.2% [53.1–73.4%]	58.6% [48.3–69.0%]
Sensitivity for Ct ≤ 33	67.9% [57.7–78.1%]	63.0% [52.4–73.5%]
for Ct ≤ 25	89.8% [81.3–98.3%]	89.8% [81.3–98.3%]
for Ct ≤ 23	91.1% [82.8–99.4%]	93.3% [86.0–100%]

Sensitivity and specificity and their 95% confidence interval are given in the table. Sensitivity depending on ORF1ab RT-PCR Ct values is indicated for the 3 categories of Ct: Ct ≤ 23, Ct ≤ 25, and Ct ≤ 33. RT-PCR is considered as the gold-standard method. Ct: crossing threshold; QAT: quantitative antigen test; RAT: rapid antigen test.

**Table 2:** QAT and RAT results for N antigen detection in dilutions of SARS-CoV-2 variants cell culture supernatants.

SARS-CoV-2 Strain *	Viral Dilutions	Qualitative QAT Results ^¤^	Quantitative QAT TCID_50_/mL ^$^	Qualitative RAT Results ^¤^
Historical B.1 lineage	No dilution	P	27,757 (71)	P
	1:10	P	2523 (13)	P
	1:100	P	270 (4)	P
	1:1000	N	31 (4)	N
	1:10,000	N	<22 (NA)	N
Alpha variant	No dilution	P	78,149 (140)	P
	1:10	P	6170 (41)	P
	1:100	P	771 (17)	P
	1:1000	N	94 (2)	N
	1:10,000	N	<22 (NA)	N
Beta variant	No dilution	P	64,402 (168)	P
	1:10	P	4773 (59)	P
	1:100	P	557 (19)	P
	1:1000	N	72 (5)	N
	1:10,000	N	<22 (NA)	N

^¤^ The qualitative results of QAT and RAT are expressed as “P” for positive and “N” for negative. ^$^ The quantitative results of QAT correspond to the mean (standard deviation) of two replicates of the undiluted and diluted cell culture supernatant. As recommended by the manufacturer, viral nucleocapsid (N) antigen value ≥200.0 TCID_50_/mL is considered as a “positive” result, between 100.0 and 200.0 TCID_50_/mL as an “equivocal” result, and <100.0 TCID_50_/mL as a “negative” result. * B.1.1.7 (Alpha), B.1.351 (Beta) are considered as variants of concern (VOC). NA: not applicable; QAT: quantitative antigen test; RAT: rapid antigen test.

## Data Availability

All other data are available from S.B. upon reasonable request.

## Supplementary Materials

The following supporting information can be downloaded at: https://www.mdpi.com/article/10.3390/diagnostics12010104/s1, Figure S1. Spearman correlation of the RT-PCR Ct value for S and ORF1ab viral genes.
