# Supplementary material for: Comparison of Rapid and Automated Antigen Detection Tests for the Diagnosis of SARS-CoV-2 Infection
Source: Diagnostics (Basel). 2022 Jan 4;12(1):104. doi: 10.3390/diagnostics12010104 (PMC8775255; doi:10.3390/diagnostics12010104)
Supplement: Supplementary file 1 [file diagnostics-12-00104-s001.zip › diagnostics-1520256-supplementary.pdf]

Supplemental data: Comparison of rapid and automated antigen detection tests for the diagnosis of SARS-CoV-2 infection

Dorian Petonnet[1], Stéphane Marot[1,2], Isabelle Leroy[1], Julien Cohier[1], Charline Ramahefasolo[1], Safietou Mansaly[1], Vincent Calvez[1,2], Anne-Geneviève Marcelin[1,2], Sonia Burrel[1,2,*]

Supplemental Figure S1. Spearman correlation of the RT-PCR Ct value for S and ORF1ab viral genes

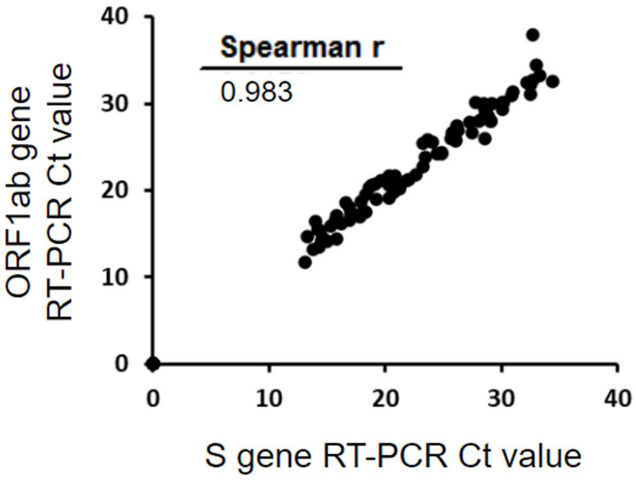

ORF1ab RT-PCR Ct value is represented on the ordinate and S RT-PCR Ct value represented on the abscissa. Correlation coefficient r calculated with Spearman formula is shown on the graph. Ct: crossing threshold.
